# Effect of Sequential Fermentation with *Lachancea thermotolerans*/*S. cerevisiae* on Aromatic and Flavonoid Profiles of Plavac Mali Wine

**DOI:** 10.3390/foods12091912

**Published:** 2023-05-07

**Authors:** Ana Mucalo, Irena Budić-Leto, Goran Zdunić

**Affiliations:** Institute for Adriatic Crops and Karst Reclamation, Put Duilova 11, 21000 Split, Croatia; irena.budic-leto@krs.hr (I.B.-L.); goran.zdunic@krs.hr (G.Z.)

**Keywords:** spontaneous fermentation, Plavac Mali, wine quality, flavonoids, anthocyanins, terpenes, esters

## Abstract

In this study, the effects of sequential fermentation of *Lachancea thermotolerans*/*S. cerevisiae* on the production of Plavac Mali wines were investigated in comparison with the commonly used inoculation of the commercial *Saccharomyces cerevisiae* strain and spontaneous fermentation. A total of 113 aroma compounds and 35 polyphenolic compounds were analyzed. Sequential inoculation resulted in a decrease in alcohol content and pH (up to 0.3% *v*/*v* and 0.12 units, respectively) and an increase in total acidity (0.6 g/L, expressed as tartaric acid). The wines produced by spontaneous fermentation exhibited the greatest diversity of volatile compounds and the highest concentration of C13 norisoprenoids, lactones, and other compounds. These wines exhibited maximum hydroxycinnamic acids, prodelphinidin monomer units, epigallocatechin, B1, B3, and B4 dimers, and total flavan-3-ols. Sequential inoculation decreased the content of the aromas and polyphenols in the wines. The practical significance of this procedure lies in the selective effect on aroma compounds, the decrease in green aromas, undetectable volatile phenols, and the decrease in bitter and astringent compounds such as gallic acid, flavan-3-ol monomers (catechin and epicatechin), and dimers (B1, B2, B3, and B4). This work demonstrates the potential of sequential and spontaneous fermentation to improve the aromatic characteristics and overall quality of Plavac Mali wines.

## 1. Introduction

Spontaneous fermentation is traditionally one of the simplest and most common ways to conduct alcoholic fermentation. Naturally present in vineyards, on berry surfaces, and on winemaking equipment, yeasts are an important source of metabolites associated with the authenticity of *terroir* [1,2,3]. Non-*Saccharomyces* yeasts include *Hanseniaspora* sp., *Debaryomyces* sp., *Pichia* sp., *Metschnikowia* sp., *Issatchenkia* sp., *Torulaspora* sp., *Candida* sp., *Kloeckera* sp., *Meyerozyma* sp., *Saccharomycodes* sp., and *Zygosaccharomyces* sp. [1,4]. The biodiversity of yeasts in vineyards is cultivar and vintage-specific [2]. The health status and ripeness of berries, the use of pesticides, and numerous other influences affect yeasts and make the practice of spontaneous fermentation unpredictable [5]. The vitality, fitness, and dominance of specific strains during fermentation depend on wine matrix composition, anaerobiosis, temperature, nutrients, and the presence of other microorganisms [6,7,8].

Non-*Saccharomyces* yeasts are active in the first phase of fermentation, while in the final stages are entirely overcome by the activity and number of *S. cerevisiae* yeast cells [3]. The slower fermentation start-up of spontaneous fermentations and yeast paradox can lead to wine spoilage and oxidation. The slow or sluggish fermentation, high residual sugar, and unwanted byproducts can cause off-flavors in final wines [9]. In the natural microbiome, the most well-adapted wine spoilage yeast specie *Brettanomyces*/*Dekkera bruxellensis* produce volatile phenols and cause loss of freshness and fruitiness of red wines. Volatile phenols are carriers of defect, animal-like notes with a low odor detection threshold of 0.047 mg/L for 4-ethylguaiacol and 0.23 mg/L for 4-ethylphenol [10]. 

The trend of inoculation with *Saccharomyces cerevisiae* strain enables carefully controlled fermentation but increases the uniformity of the wines [9]. The application of commercially available non-*Saccharomyces* yeasts in co-inoculation and sequential inoculation with *Saccharomyces cerevisiae* lately become an interesting strategy for the improvement of wine complexity [11,12,13]. The sequential inoculation of non-*Saccharomyces*/*Saccharomyces* enhances the color properties of Tempranillo and Sangiovese wines [14,15]. One of the most promising yeasts for warming climates is *Lachancea thermotolerans* known due to its ability to convert one part of sugar to lactic acid in the fermentation process. Moreover, in sequential inoculations, this strain led to a reduction in pH for 0.5 units and 0.9% in alcohol content of Merlot wines [11,13]. *L. thermotolerans* reduces volatile acidity, degrades malic acid, and increases glycerol and the concentration of higher alcohols (particularly 2-phenylethanol) and acetate and ethyl esters in wine (ethyl lactate, ethyl acetate, ethyl phenylacetate, isoamyl acetate, and isobutyl acetate) [7,11,13]. 

The sequential *Lachancea* spp./*S. cerevisiae* fermentations generate lower levels of monoterpenes in synthetic and must of Muscat of Alexandria compared to that of *S. cerevisiae* [16]. This is associated with the lack of enzymes that precede the activity of *β*-glucosidase and prevent their release from complex forms [17]. In addition, early lysis of cells of *L. thermotolerans* in sequential fermentation release of enzymes that lead to the hydrolysis of monoterpenes in monoterpene oxides and diols [16]. An increase in color intensity, formation of polymeric pigments, and polymerization indexes despite a reduction in total anthocyanins after sequential *L. thermotolerans*/*S. cerevisiae* fermentation was reported in the white wine model with the addition of malvidin-3-*O*-glucoside and flavanols [18]. The increase in color intensity after sequential fermentations could be related to a decrease in pH, a shift of anthocyanin molecules in flavylium ion, and stronger protective power of SO_2_. Higher *p*-coumaroyl glucoside content of cyanidin, petunidin, and malvidin in sequential fermentations is associated with a lower anthocyanin absorption by *L. thermotolerans* strain [19]. 

Late ripening red variety Plavac Mali is the most important Croatian grape variety, grown along Adriatic Coast and on islands. It is widely used for the production of mono-varietal red wines. The flavor and aroma of local wines are often dominated by high alcohol and pH that leads to a lack of freshness, a decrease in aroma and flavor intensity, an increase in perception of hotness, body, and viscosity, and, overall, microbial spoilage and physicochemical instability of these wines. In premium quality wine production, interventions such as the addition of tartaric, malic, lactic, or citric acid or the use of cation exchangers as electrodialysis with bipolar membranes to adjust the pH are strictly forbidden. The use of wines of early harvest dates that are dominated by fresh vegetal character, acidity, and lower color saturation [20], often less balanced, with a higher bitterness [21], is incompatible with the production of great *terroir* wines in blending [22]. Previously, a positive impact of yeast selection of *Sacharomyces cerevisiae* on dry extract, total acidity, as well as malic and succinic acid and wine color intensity of Plavac Mali wines from the islands of Vis and Korčula, was reported, especially Lalvin ICV D21 [23]. 

The objective of this research was to study the impact of three types of fermentation (sequential with *L. thermotolerans/S. cerevisiae*, conventional fermentation by *S. cerevisiae,* and spontaneous fermentation) on the aromatic and polyphenolic compounds of Plavac Mali wines, the most representative variety in Croatia. Thus far, there has been no detailed research on the subject of spontaneous fermentations and the use of non-*Saccharomyces* over conventional *Saccharomyces* yeasts, especially from this variety in which high sugars and lack of acidity represent the main problem. 

## 2. Materials and Methods

### 2.1. Environmental Conditions and Vineyard Design

The experiments with *cv*. Plavac Mali were carried out on germplasm repository vineyard located in Central and South Dalmatia wine subregion, Split (43°30′13.96″ N 16°29′56.467″ E, 14 m a.s.l.). The climate in the experimental area is Mediterranean, with hot and dry summers and rainy winters. According to Winkler index [24], in 2020, it was 2572.30 °C. This is the warmest subregion in Croatia, belonging to zone CIII [25]. The 15-year-old vines were grafted onto Boerner (*V. riparia* × *V. cinerea*) rootstock and trained to spur pruned bilateral cordon. Eight winter buds per vine were used. Spacing between vines and within rows was 2.0 m × 1.0 m (planting density 5000 plants/ha). The soil in the vineyard is brown on limestone, and rows follow north–south orientation. The vines had a vertical shoot position. 

### 2.2. Fermentation Trials

In total, 1350 kg of grapes were manually harvested on 21 September 2020 based on historical data of site and transferred to experimental winery. The grapes were destemmed and crushed. To prevent oxidation and growth of indigenous bacteria’s potassium–metabisufite to ensure 50 mg/L of sulfur dioxide (SO_2_) was added into the entire crushed mass. This initial grape must have 25.17 ± 0.06 °Brix, 3.72 ± 0.02 pH, and total acidity of 5.10 ± 0.03 expressed as g/L of tartaric acid. After homogenization, solid-liquid mass was evenly divided using balance (Sartorius, Gottingen, Germany) into three 150 L stainless steel fermenters per each of three individual fermentation trials.

#### Yeast Species

The commercial non-*Saccharomyces* active dry yeast *Lachancea thermotolerans*, strain Kt 421 (Viniflora^®^ CONCERTO™, Chr. Hansen, Hoersholm, Denmark), was used in sequential inoculation with *Saccharomyces cerevisiae* (SIHA^®^, Active Yeast 10, Eaton, Langenlonsheim, Germany) (L). Rehydration of 25 g/100 L of *L. thermotolerans* was performed in unchlorinated tap water in ratio 1:10 at temperature 25 °C, according to manufacturer’s instructions. Unsulfured grape must be added to yeast suspension in ratio 1:3, and after 20 min of acclimatization *L. thermotolerans*/must mixture was added in tanks. Addition of *Saccharomyces cerevisiae* followed 24 h after *L. thermotolerans*. Fermentative control trial was *Saccharomyces cerevisiae* (SIHA^®^, Active Yeast 10) (S). The 30 g/100 L of *S. cerevisiae* was added to warm water at 35 °C, gently stirred, and rehydrated for 20 min. Suspension was gradually acclimatized with addition of wine must be before inoculation. Spontaneous fermentation (N) was performed by yeasts naturally present in grapes. All fermentation trials were conducted in triplicate. The fermentation type used for wine production as well as their respective strains, suppliers, and fermentation conditions, are shown in Table 1. 

After completion of the primary aerobic phase of alcoholic fermentation, the wines were pressed off and decanted into 20 L wine glass balloons. The anaerobic completion of fermentation lasted three weeks. After this time, the wines were decanted, and 30 mg/L of SO_2_ was added. The second decantation of the wines took place after two months when the samples were bottled in 750 mL bottles. Wine samples were analyzed in April 2021.

### 2.3. Analysis of Standard Components of Wine 

Standard physicochemical oenological parameters of the wine were determined according to Official Methods of Wine and Must Analysis methods [26] in a laboratory accredited according to HRN ISO/IEC 17025 and are listed in Table 2. 

### 2.4. Analysis of Volatile Compounds by GCMS

Volatile compounds analysis was carried out using TRACETM 1300 Gas Chromatographer coupled to ISQ 7000 TriPlus quadrupole mass spectrometer (Thermo Fisher Scientific Inc., Bartlesville, OK, USA) equipped with TG-WAXMS A capillary column (60 m × 0.25 mm × 0.25 µm film thickness; Thermo Fisher Scientific, Bartlesville, OK, USA). Solid Phase Microextraction Arrow used for isolation of analyte was carried out using RSH Triplus autosampler (Thermo Fisher Scientific Inc., Brookfield, MO, USA). Prior to extraction, the Arrows were preconditioned in the injector port of the gas chromatograph according to the manufacturer’s instructions. The SPME Arrow procedure was performed according to [27] with fibers (Supelco, Bellefonte, PA, USA) coated with stationary phase and film thicknesses as follows: divinylbenzene–carboxen–polydimethylsiloxane 50/30 μm (DVB/CAR/PDMS) Arrow system. A total of 5 mL of wine and 2.5 g of NaCl salt were added to the glass vials closed with polytetrafluoroethylene (PTFE) coated silicone rubber septum. Equilibration of the wine sample was performed at 55 °C for 10 min. Adsorption of the analyte was performed at 55 °C for 60 min. Desorption was performed in a liquid chromatograph injector at 250 °C for 7 min. The volatile compounds injected into the inlet were delivered to the column at a splitless mode, and helium was used as a carrier gas at a constant flow rate 1 mL/min. Chromatographic analysis was performed at temperature program in the range of 40 to 210 °C. Mass spectrum recording was performed by monitoring the current of all ions in the range of 20 to 500 *m*/*z* while the ionization energy was 70 eV. The data obtained were processed using ChromeleonTM Data System (Thermo Fisher Scientific Inc., Bartlesville, OK, USA). Identification of volatile compounds was performed by comparing the recorded mass spectrum with the data available in Wiley Registry 12th Edition/NIST (National Institute of Standards and Technology) Mass Spectral Library and the calculation of the retention index of the analytes. The Retention index (RI) was calculated using alkane standards C8–C20 (Sigma Aldrich, St. Loius, MO, USA) as described [27,28]. 

### 2.5. Analysis of Polyphenolic Compounds by HPLC

A high-performance liquid chromatography (HPLC) system with diode array (DAD) and fluorescence detector (FLD) (Agilent 1100, Palo Alto, CA, USA) was used for the identification and quantification of the polyphenolic compounds in wines according to [29]. A wine sample preparation included filtration through a 0.22 μm PTFE membrane filter (Miliford, MA, USA) in the vial of the autosampler. Chromatographic separation of analytes was performed on a Luna phenyl-hexyl column (Phenomenex, Torrance, CA, USA) (250 mm × 4.6 mm i.d., 5 μm particle size) with a phenyl guard column (4.0 mm × 3.0 mm) (Phenomenex, Torrance, CA, USA), tempered at 50°C. A volume of 20 μL was injected into the HPLC system. Two mobile phases were used as gradient solutions for elution: (A) water/phosphoric acid (99.5:0.5, *v*/*v*) and (B) acetonitrile/phosphoric acid/water (50:49.5:0.5, *v*/*v*/*v*). The flow rate of the mobile phase was 0.9 mL/min. Detection of flavonoid compounds was performed using a DAD detector at different wavelengths: flavonols at 360 nm and anthocyanins at 518 nm. Flavan-3-ols were quantified with a more sensitive fluorescence detector at the wavelength of excitation λex = 225 nm and the wavelength of emission λem = 320 nm. Peak identification of each flavonoid eluate compound was performed by comparing the retention times, DAD, and fluorescence spectrum with the external standards according to the method [29,30]. The quantitative values of the identified compounds were calculated using the calibration curves and the peak area of the corresponding standard compounds prepared by the analysis of the external standards and expressed in mg/L.

### 2.6. Statistical Analyses 

One-way analyses of variance (ANOVA) and mean separation using Stats-Fisher’s LSD test were performed to determine differences among the three different fermentation trials. Analyses of variance for the data were performed in SAS (SAS Institute, Inc., Cary NC, USA). Results for all parameters are expressed as mean ± standard deviation. All reported uncertainties are the standard deviations of three replicates of a treatment. Aroma and polyphenolic dataset were separately subjected to principal component analysis (PCA), illustrating association between L, S, and N wine samples and selected compounds in PC1 and PC2. 

## 3. Results and Discussion

### 3.1. Physiochemical Composition of the Wines Produced by Sequential L, Conventional S and Spontaneous N Fermentation 

The physicochemical properties of nine Plavac Mali wines produced using *Lachancea thermotolerans* sequential-inoculated with *Saccharomyces cerevisiae*, *Saccharomyces cerevisiae*, and spontaneous fermentation are shown in Table 2. Wines fermented by the L yeast strain sequentially inoculated with S had a lower alcohol content of 14.93 ± 0.06% *v*/*v* in comparison to those obtained by N of 15.23 ± 0.12% *v*/*v*. L wines had a significantly higher total acidity of 5.9 ± 0.00 expressed as g/L of tartaric acid. The pH of L wines was significantly lower only when compared to N wines. A similar decrease in alcohol content and pH was reported in Merlot wines produced by different inoculations by *Lachancea thermotolerans* and *Saccharomyces cerevisiae* [11]. However, the reducing sugar amount of 7.13 ± 0.81 g/L indicated the production of semi-dry Plavac Mali wines by using the L strain.

### 3.2. Aroma Profile of the Plavac Mali Wines Produced by Sequential L, Conventional S and Spontaneous N Fermentation

**Figure 1:**
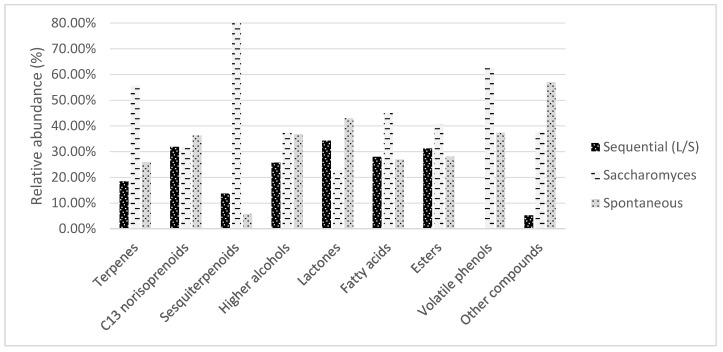
Relative abundance of groups of volatile compounds quantified in wines produced by sequential (*Lachancea thermotolerans*/*S. cerevisiae*), conventional *Saccharomyces cerevisiae* and spontaneous fermentation.

One hundred thirteen aroma compounds were identified and quantified using GC-MS analysis in wines of Plavac Mali produced by three types of alcoholic fermentation (Table 3). Aroma compounds are grouped into nine chemical classes of terpenes, C13 norisoprenoids, sesquiterpenoids, higher alcohols, lactones, esters, fatty acids, volatile phenols, and other compounds, as shown in Figure 1. The ANOVA results showed significant differences (*p* < 0.05) for the 108 aroma compounds between wines obtained by different fermentations. 

#### 3.2.1. Varietal Aroma Compounds in the Plavac Mali Wines Produced by Sequential L, Conventional S and Spontaneous N Fermentation 

The differences between wines produced with three fermentations (L, S, and N) in varietal aromas, terpenes, and C13 norisoprenoids were quantitative and qualitative in nature. Wines produced by *Saccharomyces cerevisiae* had a significantly higher concentration of individual and total terpene compounds, except *α*-terpinene, *β*-myrcene, and *trans*-linalool oxide (furanoid), the maximum of which was in N wines. L wines had the lowest concentration of each individual and total terpene compound, except citronellol acetate only detected in those wines. The type of fermentation had a significant impact on the individual and total sum of C13 norisoprenoids in produced wines. N wines had the highest total sum of norisoprenoids. Wines of three fermentations significantly differed in the concentration of TPB and 2,3-dehydro-4-oxo-*β*-ionol. The highest concentration of TPB was in N wines and 2,3-dehydro-4-oxo-*β*-ionol in L wines. N wines differed from S wines in significantly lower concentrations of *β*-damascenone and higher of 2,5,8-trimethyl-1,2,3,4-tetrahydro-1-naphthalenol. In wines, those varietal aromas exist as glycosidically bound precursors that are released during fermentation by yeast enzyme hydrolyzation [31,32]. Yeasts differ in the capability of the detachment of those compounds in dependence on the chemical structure of the sugar and the aglycone moieties [33]. Acid hydrolysis plays an important role in the rearrangements of terpenes and C13 norisoprenoids. Although, a small part of de novo biosynthesized terpene compounds by *Saccharomyces cerevisiae* have been reported in Cabernet Sauvignon [34,35]. 

#### 3.2.2. Fermentation Aroma Compounds in the Plavac Mali Wines Produced by Sequential L, Conventional S and Spontaneous N Fermentation

Among fermentation aromas, L wines had significantly lower concentration of 11 individual (3-methyl-1-butanol, 1-decanol, 1-hexanol, 1-nonanol, 1-octanol, 1-octen-3-ol, 1-pentalon, 4-methyl-1-pentanol, 3-penten-1-ol, 2-phenylethanol, *trans*-3-hexen 1-ol) and total sum of higher alcohols. The most abundant higher alcohol in all wines was a 3-methyl-1-butanol (isoamyl alcohol), followed by 1-decanol and 1-hexanol in S and N wines. The 3-methyl-1-butanol and isobutanol have a strong sensory effect on wine [36,37]. In all wines, the concentration of 3-methyl-1-butanol exceeds its odor detection threshold of 30000 µg/L [38,39], which provides fresh and jammy fruit and butyric aroma notes [36]. The L wines had the lowest concentration of 3-methyl-1-butanol. A decrease in 3-methyl-1-butanol was previously found in red Sangiovese and in white Emir wines when produced by *L. thermotolerans*/*S. cerevisiae* co-inoculation or by pure *L. thermotolerans* in comparison to pure *S. cerevisiae* [13,40]. The L wines, when compared to S and N, show an unexpected increase in isobutanol and a decrease in 2-phenylethanol that could be related to *L. thermotolerans* strain-derived differences [11]. The sequential inoculation *L. thermotolerans/S. cerevisiae* led to a significant decrease in grape-derived higher alcohols 1-hexanol and *trans*-3-hexen-1-ol, carriers of green aromas [41]. 

Among 36 detected esters carriers of fruity and floral aromas in the wine, 19 of them were ethyl esters, followed by methyl and acetate esters. S wines had maximum values of 11 individuals and a total sum of esters. The predominant was 2-methyl-propanoate concentration, which was significantly higher in L wines than in others, 4002.09 ± 92.78 µg/L. The 2-methyl-propanoate is a carrier of a sweet fruity, rum-like odor [39]. L wines contained significantly higher concentrations of ethyl butanoate and ethyl-2-hydroxypropanoate (ethyl lactate). N wines had the highest diversity of esters that can be associated with high biodiversity of the spontaneous non-*Saccharomyces* population initially must be able to produce a greater variety of extracellular enzymes than *S. cerevisiae* [42]. However, N wines had the lowest total sum of esters at 7690.21 ± 61.69 µg/L; among them, 2-phenylethyl acetate (rose, honey, tobacco) predominate was 1008.36 ± 15.32 µg/L. While in L wines, the concentration of this ester was a minimal 642.87 ± 11.88 µg/L. Previously, a maximum of 2-phenyletyl acetate was found in un-inoculated and sequential inoculated (*L. thermotolerans* strain four and *Saccharomyces cerevisiae*) Merlot wines [11]. A similar mismatch is seen in the concentration of diethyl butanoate, a carrier of fruity and caramel aromas. In Plavac Mali wines maximum of this compound was in N wines, and the minimum in S wines, contrary to data previously reported [33]. Differences between esters are related to a yeast strain but also to grape juice components [35] and juice composition as sugar content, YAN levels, amino acid profile, carbon and nitrogen content, temperature, and aeration degree during vinification [6,43]. Only in S wines was ethyl heptanoate detected. Critical precursors of this compound are amino acids threonine and alanine [44]. It could be that strains mutually differ in preferred nitrogen sources, as alanine is easily incorporated into the cell’s metabolic pathways, while threonine has a great impact on *Saccharomyces* biomass production, exponential growth rate, and fermentation time [45]. The L, S, and N wines show different relationships between esters and their higher alcohol precursors. The L and N wines have a higher amount of alcohol precursor in comparison to ester, as in the case of ethyl-2,4-hexadienoate, which could be associated not only with a lower activity of ethanol hexanoyl transferase enzyme but also with a specific higher preference for this enzyme to a specific substrate, such as octanoyl-CoA [46]. Similar is seen in the concentration of 1-hexanol and hexyl acetate in N wines. 

S wines had a significantly higher concentration of four fatty acids as follows, 3-methylbutanoic, hexanoic, nonanoic, and octanoic acid, and 4-hydroxy-butanoic acid was only detected in those wines. The total sum of fatty acids followed the same pattern as the most abundant among them, 3-methylbutanoic acid, maximum in S (14,821.45 ± 335.48 µg/L) and minimum in N (8885.76 ± 122.63 µg/L) wines. An increase in hexanoic, octanoic, and decanoic acid and ethyl esters of those acids by *Saccharomyces cerevisiae* wine strain is correlated to a higher nitrogen concentration of must and to a specific consumption pattern of this yeast in monoculture and co-culture fermentations [47,48]. Those fatty acids are associated with a sweet, rancid, and cheesy aroma note in wines. In our research, in all wines, only two of them, butanoic and hexanoic acid, were in concentrations higher than the odor detection threshold (173 and 420 µg/L, respectively) [49]. The L wines had a significantly higher concentration of 2,4-hexadienedioic, butanoic, decanoic, and dodecanoic acid in comparison to S wines and 3-methylbutanoic and butanoic acid in comparison to N wines. In L and N wines, the higher diversity between fatty acids and increase in fatty acids, higher alcohols, and esters could be due to a specific metabolic interaction between *L. thermotolerans* and *S. cerevisiae* [6] and non-*Saccharomyces* and *S. cerevisiae* in comparison to monoculture. 

The N wines had the highest concentration of the total sum of lactones (933.80 ± 32.44 µg/L), and the S wines were minimal (495.39 ± 11.85 µg/L). In L and N wines, a higher concentration of butyrolactone over *γ*-heptalactone is seen, while in S wines, *γ*-heptalactone predominates. Lactones are formed during fermentation from fatty acids in *the β*-oxidation pathway [50], although some of them have been found in the over-ripe grape of Syrah [51]. Previously, the most abundant lactone in wines produced with different *Saccharomyces* strains (three *S. cerevisiae* and one *S. bayanus*) was butyrolactone [33]. 

The S wines had a maximum of volatile phenols (113.94 ± 2.09), with a maximum of 4-ethyl guaiacol and 4-ethy phenol, while none of the volatile phenols was detected in L wines. Differences in volatile phenols in *S. cerevisiae* compared to non-*Saccharomyces*-produced wines were previously reported [52]. Production of volatile phenols is associated with a non-oxidative decarboxylation of the hydroxycinnamic acids early in fermentation steps after four to 48 h [10,53]. In red wines, enzyme activity is inhibited by catechin flavan-3-ol monomers and dimers [10]. It could be that in our case, *L. thermotolerans* blockade this process early in fermentation steps, as the decarboxylase activity of *S. cerevisiae* strain is maximal at the beginning of the stationary phase and decrease rapidly when the viability of the cells diminishes [10]. Those two compounds below 140 and 620 µg/L for 4-ethyl guaiacol and 4-ethyl phenol, respectively, positively contribute to red wine aroma [54]. In higher concentrations, those are carriers of barnyard, animal, and clove-like and smoke aromas associated with a “Brett character” found in wines contaminated with *Brettanomyces* yeast [55]. Among other compounds, the most abundant was Methionol (3-(methylthio)-1-propanol), which was especially abundant in N wines (5978.68 ± 107.43 µg/L) contrary to minimal concentrations in L wines (53.23 ± 3.53 µg/L). This volatile sulfur flavor compound is derived from the amino acid methionine. It has a low odor threshold ranging from about one to 3 ppm (1000–3000 µg/L) and perception of baked cabbage [56]. Production of this compound depends on the methionine level in the yeast strain used and can be modulated by quantity and quality of nitrogen nutrient selection [43]. 

### 3.3. Polyphenolic Profile of the Plavac Mali Wines Produced by Sequential L, Conventional S and Spontaneous N Fermentation

Both sequential L and conventional S fermentation led to a significant reduction in non-flavonoid HCA acids, mainly the most predominant one, caftaric acid (Table 4). In berries caftaric, coutaric, and fertaric acids are tartaric esters of HCAs. In fermentation, those are released by the cinnamyl esterase enzymes and can be transformed by hydroxycinnamate decarboxylase and vinylphenol reductase into volatile phenols. In S wines significant decrease in HCA and caftaric acid and an increase in volatile phenols is associated with a strong hydroxycinnamate decarboxylase activity of *S. cerevisiae* [10]. In L wines, despite a significantly lower concentration of HCA and caftaric acid compared to N wines, there were no detectable volatile phenols in wines. It could be that in sequential inoculations, *L. thermotolerans* early in fermentation use those precursors in other reactions and blockade the production of those *off* flavors from *S. cerevisiae* [10,54]. In addition, those volatile phenols can react with anthocyanins and form stable fermentation products vinylphenolic pyranoanthocyanins. 

L wines have significantly lower concentrations of gallic, syringic acid, and total hydroxybenzoic acids. It could be that lower temperatures, kinetic of phenolic compound extraction, and kinetic of fermentation (slower rate of sugar degradation) at the start and lower alcohol content in L wines decrease extraction of gallic acid from grape seeds [20]. Other hypotheses are that already extracted gallic acid during fermentation reacts with alcohols formed by yeasts [57] or is adsorbed by the cell wall [58]. Previously, a selection of *S. cerevisiae* strains in Plavac Mali wine production had an impact on this HBA with a maximum of S16 strain [23]. Moreover, an increase in S and N wines can be associated with a tannase activity of *S. cerevisiae* or one of the yeasts from the natural population that can biodegrade hydrolyzable tannins [59]. A decrease in phenolic acids in L wines can lead to a higher sensory quality of those wines, as those are known to contribute to the astringency and bitterness of wine and have a synergetic effect on the perception of astringency [60].

Sequential L fermentation led to a significant decrease in the concentration of eight individual (four individual anthocyanin-3-*O*-glucoside as follows delphinidin, petunidin, peonidin, malvidin), and acetyl, caffeoyl, and *p*-coumaroyl derivatives of malvidin-3-*O*-glucoside, peonidin-3-*O*-glucoside *p*-coumaroyl and the total sum of anthocyanins. The greatest reduction is seen in the main anthocyanin compound, malvidin-3-*O*-glucoside, the concentration of which in S wines was 85.30 ± 2.80 mg/L, and in L wines decreased to 70.32 ± 0.69 mg/L. S and N wines differed only in the concentration of delphinidin-3-*O*-glucoside and malvidin-3-*O*-caffeoyl-glucoside, both of which reached a maximal value in N wines. Although a decrease in anthocyanins in L wines could be associated with a stronger adsorption capacity of the yeast cell wall [61]. Previously, higher cyanidin-3-*O*-(6″-*p*-coumaroylglucoside), petunidin-3-*O*-(6″-*p*-coumaroylglucoside), and malvidin-3-*O*-(6″-*p*-coumaroylglucoside) content in sequential fermentations is associated to a lower anthocyanin absorption by *L. thermotolerans* strain [19]. More probably, those anthocyanins react with pyruvic acid and acetaldehyde and form more stable compounds such as vitisin, acetylvitisin, and *p*-coumaroylvitisin A and B [61]. Another explanation could be that certain differences in wine matrix are partially caused by an increase in total acidity and a decrease in pH initiate reactions between hydroxycinnamic acids and anthocyanins and the formation of vinylphenolic pyranoanthocyanins without the enzymatic support of yeast [62]. Among flavonols, the greatest reduction is seen in sequential L fermentations, with a significant decrease in miricetin-3-*O*-glucoside, quercetin-3-galactoside, quercetin-3-*O*-glucoside, kaempferol-3-glucouronide, and total flavonols. The S and N wines differed significantly only in 3-*O*-glucouronides of miricetine and quercetine, with the highest concentrations in N and S wines, respectively. Flavonols react with anthocyanins or each other, the result of which are anthocyanin–flavanol, flavanol–anthocyanin, and flavanol–flavanol adducts. The main yeast-derived byproducts that participate in those processes are acetaldehyde and pyruvic acid. It could be that larger differences between L and S than between N and S are the result of the interrelationships between these two yeasts. *L. thermotolerans* is known as the strain that generally produces lower concentrations of acetaldehyde and pyruvic acid, but this is highly influenced by a strain and used inoculation modalities (sequential or co-inoculation) [11]. Sequential L fermentation led to a significant decrease in catechin, epicatechin, proanthocyanidin dimers B1, B2, B3, B4, and the total sum of flavan-3-ols in comparison to S and N wines. Those flavan-3-ols, especially catechin and epigallocatechin, are indicators of seed and skin extraction in wine [63]. There are scarce results in the literature about the impact of yeasts on flavan-3-ol monomer and dimer wine profile. In addition, differences between varieties in polyphenolic composition and extractability of those compounds impact the final signature of the yeast on wine. The addition of catechin in wines has a strong impact on the direction of condensation reactions by yeasts [18]. The highest concentration of epigallocatechine, proanthocyanidin B1, B3, and B4, and flavan-3-ols was in N wines. The significant reduction in epigallocatechin was in S wines, while catechin and epicatechin were the same as in N wines. *S. cerevisiae* strain S16 was previously reported to produce higher concentrations of catechin, epicatechin, and epigallocatechin in wines of Plavac Mali [23].

### 3.4. PCA Analysis of Aroma and Polyphenolic Plavac Mali Wine Composition Produced by Sequential L, Conventional S and Spontaneous N Fermentation

**Figure 2:**
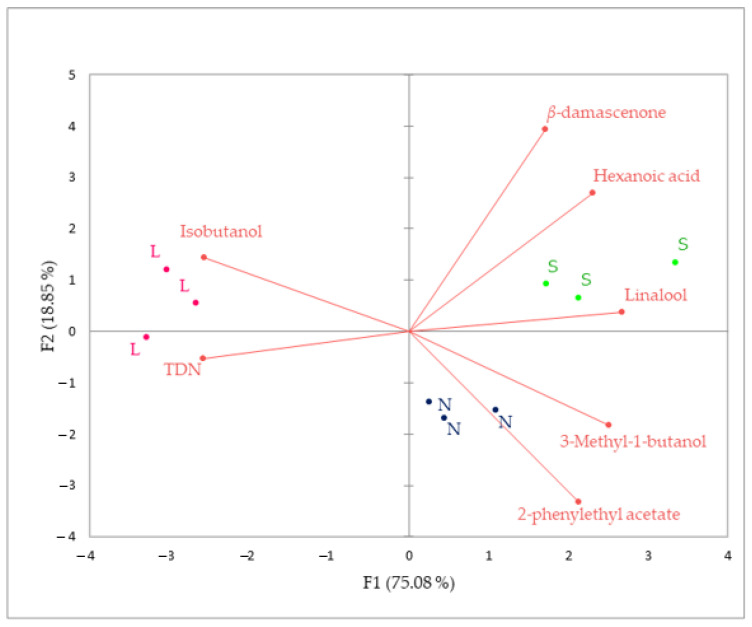
PCA analysis of aroma compounds of Plavac Mali wines produced by sequential L, conventional S and spontaneous N fermentation.

The contribution of F1 was 75.08%, and of F2, 18.85% (Figure 2). According to the PCA, wines of different treatments were clearly separated. Wine sample replicates grouped together for three fermentation treatments. S and N wines were located on the positive side of PC1 and were characterized by five aroma compounds: linalool, *β*-damascenone, hexanoic acid, 3-methyl-1-butanol, and 2-phenylethyl acetate. L wines were located on the negative side of PC1 and correlated with TDN and isobutanol aroma compounds. The seven compounds were selected as the most important contributors to floral and fruity aromas (linalool, *β*-damascenone, 2-phenyl acetate) and as important enhancers and suppressors of fruity aromas (*β*-damascenone, 3-methyl-1-butanol, isobutanol), with only two from green and petroleum class, hexanoic acid and TDN [33,36,37].

**Figure 3:**
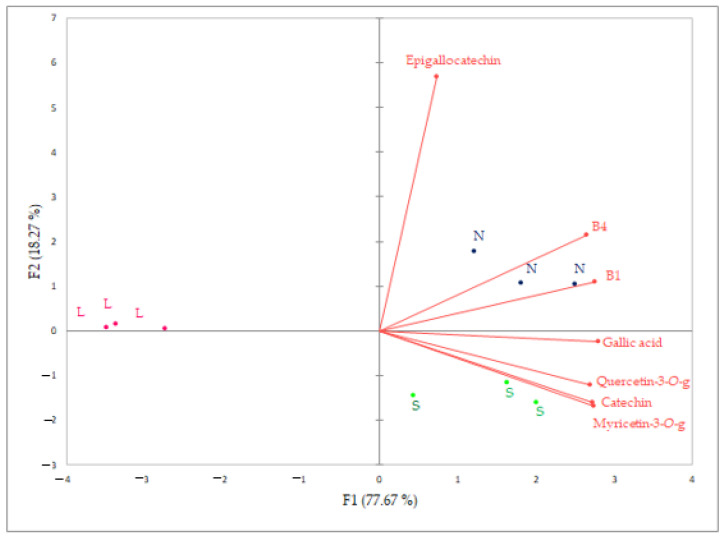
Principal component analysis bi-plot of one non-flavonoid and 6 flavonoid variables of Plavac Mali wines produced by different fermentation treatments (sequential L, conventional S and spontaneous N fermentation).

The data for one non-flavonoid (gallic acid) and six flavonoids (myricetin-3-*O*-glucoside, quercetin-3-*O*-glucoside, catechin, epigallocatechin, B1, B4) variables were processed using principal component analysis (Figure 3) to differentiate among Plavac Mali wines obtained by three different fermentations (sequential (L), conventional (S) and spontaneous (N)). The first two axes represent 95.94% of the initial variability. The first eigenvalue equals 5255 and represents 77.67% of the total variability explained by PC1. PC2 explains 18.27% of the total variability. According to the PCA, wines produced by different yeasts were clearly separated. Wine sample replicates grouped together for all three treatments. N and S wines were grouped on the positive side of PC1, in the first and fourth quadrants, respectively, and characterized by all nine variables. Sequential inoculation L wines were grouped on the negative side of PC1. 

PCA confirmed that the fermentation treatment affected the volatile and polyphenolic profiles of the wine samples.

## 4. Conclusions

The type of fermentation had an important role in defining the aroma and polyphenolic composition of Plavac Mali wines. Significant differences were estimated for the 108 aroma and 30 polyphenolic compounds identified and quantified in Plavac Mali wines of three different fermentations. Sequential *Lachancea thermotolerans*/*S. cerevisiae* fermentation led to wines with a lower alcohol content, higher residual sugar (>7 g/L), and total acidity. The L wines had significantly higher concentrations of 15 individual aroma compounds, among which higher alcohols predominate. The benefits of sequential fermentation were no detectable volatile phenols and significantly lower concentrations of higher alcohols, namely those responsible for green aromas (1-hexanol, *trans*-3-hexen-1-ol, 1-octen-3-ol) and other compounds, especially methionol. The L wines had a minimum of gallic acid, (8) anthocyanins, (4) flavonols, (7) flavan-3-ols, and the total sum of each of those compounds.

The spontaneous N wines had significantly higher concentrations of 25 individual aroma compounds, among which esters predominate. The N wines had a maximum of caftaric and total sum of hydroxycinnamic acids, delphinidin-3-*O*-glucoside and caffeoyl-glucoside of malvidin, miricetin-3-*O*-glucoside, prodelphinidin monomer unit epigallocatechin, and B1, B3 and B4 dimers and the total sum of flavan-3-ols. The results of PCA analysis showed that the first two principal components describe 93.93% of the variation among selected aromas and 95.94% among polyphenolic compounds in wines produced by different fermentation trials. S and N wines were located on the positive side of PC1 and were characterized by five aroma compounds: linalool, *β*-damascenone, hexanoic acid, 3-methyl-1-butanol, and 2-phenylethyl acetate, and seven polyphenolic compounds: gallic acid, myricetin-3-*O*-glucoside, quercetin-3-*O*-glucoside, catechin, epigallocatechin, B1 and B4. L wines were located on the negative side of PC1 and correlated with TDN and isobutanol aroma compounds. This work demonstrates the importance of sequential and spontaneous fermentation in the improvement of Plavac Mali wines. Further knowledge of yeast interactions and strain-specific data in spontaneous fermentation are needed.

## Figures and Tables

**Table 1:** Yeast species and fermentation conditions in wine production.

Fermentation	L	S	N
Yeast species	*Lachancea thermotolerans* *Saccharomyces cerevisiae*	*Saccharomyces cerevisiae*	Spontaneous yeasts
Strain	Kt 421 10	10	Unknown
Suppliers	Viniflora, Concerto Siha, Active Yeast 10	Siha, Active Yeast 10	Natural
Timing of yeast inoculation	after destemming after 24 h	after destemming	No
Temperature (°C)	25.8–26.6	26.4–26.9	24.8–25.6
Frequency of punch down	every 12 h/5 min	every 12 h/5 min	every 12 h/5 min
Duration of maceration	7 days	7 days	7 days

**Table 2:** Physicochemical composition of the Plavac Mali wines produced by sequential L, conventional S and spontaneous N fermentation.

Compound	L	S	N
Alcohol (% *v*/*v*)	14.93 ± 0.06 b	15.13 ± 0.06 ab	15.23 ± 0.12 a
Reducing sugar (g/L)	7.13 ± 0.81 a	4.73 ± 0.38 b	5.27 ± 1.27 ab
Extract (g/L)	25.43 ± 0.61 a	24.77 ± 0.95 a	25.43 ± 0.23 a
Ash (g/L)	2.81 ± 0.22 a	3.11 ± 0.01 a	2.91 ± 0.05 a
pH	3.56 ± 0.03 b	3.59 ± 0.03 b	3.68 ± 0.02 a
Total acidity (g/L as tartaric acid)	5.90 ± 0.00 a	5.60 ± 0.00 b	5.30 ± 0.10 c
Volatile acidity (g/L as acetic acid)	0.50 ± 0.00 a	0.40 ± 0.00 b	0.40 ± 0.00 b

Values are means with standard deviations of three separate repetitions. Values with different letter (a, b, c) in the same row are significantly different, according to the Fisher’s LSD test (*p <* 0.05).

**Table 3:** Volatile aroma compounds in Plavac Mali wines produced by sequential L, conventional S and spontaneous N fermentation expressed as (µg/L).

Aroma Compound (µg/L)/Yeast	L	S	N
*α*-Ocimene	nd	32.84 ± 1.33	nd
Citronellol	134.90 ± 10.47 c	657.16 ± 13.62 a	201.63 ± 4.32 b
*α*-Pinene	1.11 ± 0.12 c	8.60 ± 0.68 a	4.61 ± 0.41 b
*α*-terpinene	nd	6.29 ± 0.67 b	11.61 ± 0.21 a
*α*-terpineol	4.78 ± 0.97 c	39.29 ± 0.92 a	7.19 ± 0.29 b
D-Limonene	nd	26.84 ± 0.99 a	17.71 ± 2.03 b
Hotrienol	3.12 ± 0.16 c	8.95 ± 0.25 a	7.30 ± 0.17 b
Linalool	10.29 ± 0.30 c	37.53 ± 0.68 a	25.88 ± 3.33 b
*β*-myrcene	107.20 ± 5.32 a	95.21 ± 2.11 a	111.06 ± 9.56 a
Menthol	36.62 ± 2.07 b	43.79 ± 2.81 a	39.20 ± 0.85 ab
Citronellol acetate	5.38 ± 0.48	nd	nd
*trans*-linalool oxide (furanoid)	22.30 ± 0.82 b	26.55 ± 2.84 ab	32.65 ± 3.50 a
Ʃ Terpenes	325.71 ± 12.66 c	983.04 ± 10.15 a	458.84 ± 15.04 b
*β*-damascenone	5.81 ± 0.52 ab	6.68 ± 0.30 a	5.72 ± 0.14 b
TDN	7.27 ± 0.23 a	6.50 ± 0.24 b	6.84 ± 0.12 ab
TPB	3.32 ± 0.28 c	6.57 ± 0.20 b	8.91 ± 0.86 a
Vitispirane A	14.50 ± 0.89 b	22.87 ± 0.93 a	17.62 ± 1.06 ab
Vitispirane B	11.69 ± 0.78 b	19.36 ± 2.01 a	16.73 ± 1.26 a
2,3-dehydro-4-oxo-*β*-ionol	12.18 ± 0.49 a	nd	8.01 ± 0.14 b
4-hydroxy-*β*-ionone	5.66 ± 0.40 a	4.19 ± 0.84 b	5.45 ± 0.30 ab
2,5,8-trimethyl-1,2,3,4-tetrahydro-1-naphthalenol	5.85 ± 0.49 a	nd	6.13 ± 0.18 a
Ʃ Norisoprenoids	66.28 ± 2.83 b	66.17 ± 2.21 b	75.41 ± 0.68 a
*cis*-*α*-bisabolene	5.45 ± 0.39	nd	nd
Isocaryophyllene	15.14 ± 0.68	nd	nd
*cis*-*β*-farnesene	18.60 ± 1.13 b	231.04 ± 20.71 a	16.60 ± 0.99 b
Ʃ Sesquiterpenoids	39.19 ± 0.87 b	231.04 ± 20.71 a	16.6 ± 0.99 b
*cis*-6-nonen-1-ol	16.57 ± 0.40 b	17.57 ± 1.90 ab	20.29 ± 0.91 a
1,3-octanediol	3.08 ± 0.29 a	nd	2.76 ± 0.41 a
1-butanol	1617.69 ± 41.58 b	1386.23 ± 76.94 b	2551.74 ± 183.83 a
3-methyl-1-butanol (Isoamyl alc)	77311.14 ± 1411.88 b	114386.01 ± 4223.04 a	115538.37 ± 5480.85 a
1-decanol	5899.25 ± 113.55 c	14565.27± 300.42 a	11731.37 ± 269.77 b
2-ethyl-1-hexanol	41.21 ± 1.83 a	34.23 ± 3.93 b	nd
1-hexanol	8694.68 ± 167.31 b	13825.33 ± 1033.70 a	13957.16 ± 344.91 a
1-dodecanol	nd	8721.94 ± 153.36 a	6168.52 ± 285.03 b
1-nonanol	33.43 ± 1.18 c	60.87 ± 2.26 a	53.72 ± 1.63 b
1-octanol	72.78 ± 2.34 b	158.91 ± 4.90 a	164.51 ± 3.51 a
1-octen-3-ol	65.00 ± 1.29 b	90.10 ± 2.39 a	94.44 ± 5.72 a
1-pentanol	3347.18 ± 84.22 b	4621.36 ± 144.71 a	4605.15 ± 252.26 a
4-methyl-1-pentanol	462.46 ± 9.84 c	1994.52 ± 41.12 b	2595.56 ± 184.89 a
1-propanol	5259.57 ± 90.84 a	805.32 ± 67.88 c	1110.80 ± 128.61 b
3-ethoxy-1-propanol	1833.89 ± 11.51 a	76.67 ± 2.94 b	30.29 ± 1.09 c
3-ethyl-4-methylpentan-1-ol	4013.40 ± 89.47 a	1204.50 ± 173.69 c	1682.82 ± 58.59 b
*trans*-3-hexen-1-ol	286.54 ± 10.56 b	5268.64 ± 132.29 a	5299.20 ± 286.28 a
*cis*-3-hexen-1-ol	696.89 ± 13.14 b	692.10 ± 17.18 b	985.02 ± 43.83 a
2-heptanol	7.36 ± 0.46 b	8.02 ± 0.75 b	12.67 ± 2.33 a
2-nonanol	8.27 ± 1.21 a	9.87 ± 1.21 a	9.91 ± 0.25 a
*trans*-2-octen-1-ol	37.62 ± 2.99 a	41.62 ± 0.77 a	38.36 ± 1.06 a
3-methylpentan-1-ol	2008.78 ± 40.89	nd	nd
3-octanol	30.45 ± 0.76 b	30.01 ± 1.76 b	54.18 ± 1.15 a
3-methyl-3-pentanol	1221.41 ± 22.51	nd	nd
3-penten-1-ol	1816.10 ± 72.50 c	5797.31 ± 104.23 a	3722.42 ± 116.91 b
Isobutanol	8618.19 ± 153.22 a	7359.04 ± 430.46 b	7477.05 ± 135.44 b
2-phenylethanol	4639.36 ± 88.67 c	6299.05 ± 144.66 a	5868.90 ± 191.30 b
2,3-butanediol	1197.41 ± 21.02 a	1233.12 ± 47.03 a	811.39 ± 14.49 b
Ʃ Higher alcohols	129239.69 ± 1889.85 b	188687.6 ± 4988.08 a	184586.61 ± 7231.42 a
*trans*-oak lactone	nd	16.95 ± 1.15	nd
*γ*-heptalactone	183.47 ± 2.62 c	461.44 ± 11.83 a	391.96 ± 12.44 b
Butyrolactone	561.67 ± 10.20 a	17.01 ± 0.62 b	541.83 ± 21.09 a
Ʃ Lactones	745.14 ± 11.52 b	495.39 ± 11.85 c	933.8 ± 32.44 a
2,4-hexadienedioic	3.00 ± 0.28 b	nd	4.34 ± 0.59 a
3-methylbutanoic acid	5090.29 ± 89.57 b	7670.03 ± 49.48 a	3719.85 ± 52.49 c
Butanoic acid	768.12 ± 15.11 a	nd	300.17 ± 7.78 b
4-hydroxy-butanoic acid	nd	870.72 ± 17.53	nd
Decanoic acid	359.63 ± 8.29 b	250.41 ± 49.27 c	525.00 ± 32.10 a
Dodecanoic acid	22.15 ± 2.25 a	nd	24.30 ± 1.34 a
Hexanoic acid	1902.41 ± 33.67 b	3240.13 ± 167.50 a	2120.80 ± 102.51 b
2-Ethyl hexanoic acid	nd	nd	81.03 ± 1.52
Nonanoic acid	67.28 ± 2.53 c	167.85 ± 4.93 a	117.54 ± 2.07 b
Octanoic acid	991.91 ± 17.59 c	2622.32 ± 79.09 a	1982.15 ± 44.84 b
*trans*-2-undecenoic acid	nd	nd	10.57 ± 0.79
Ʃ Fatty acids	9204.78 ± 161.32 b	14821.45 ± 335.48 a	8885.76 ± 122.63 b
3-methyl-1-butanoate	726.90 ± 13.96 b	2781.87 ± 214.68 a	974.04 ± 61.19 b
Ethyl-2,4-hexadienoate	4.50 ± 0.52 c	11.98 ± 1.20 a	6.94 ± 0.60 b
Ethyl-2-hexenoate	3.35 ± 0.40 b	19.08 ± 1.30 a	21.24 ± 1.34 a
Diethyl-malate	68.68 ± 2.31 b	111.72 ± 10.14 a	79.49 ± 1.42 b
3-methylbuthyl decanoate	6.08 ± 0.73 b	nd	7.45 ± 0.52 a
2-phenylethyl acetate	642.87 ± 11.88 c	885.48 ± 19.53 b	1008.36 ± 15.32 a
Hexyl acetate	19.65 ± 1.32 b	48.49 ± 2.85 a	13.16 ± 0.60 c
Pentyl acetate	2.63 ± 0.10 b	nd	3.30 ± 0.23 a
Phenyl-ethyl acetate	51.89 ± 1.93 b	80.09 ± 3.83 a	83.83 ± 4.52 a
Methyl-2-hydroxybenzoate	4.36 ± 0.55 b	7.83 ± 0.65 a	2.00 ± 0.24 c
Diethyl butanoate	869.19 ± 17.30 b	99.75 ± 5.79 c	978.94 ± 70.75 a
Ethyl butanoate	42.07 ± 1.13 a	nd	34.27 ± 2.58 b
Ethyl decanoate	95.59 ± 3.88 b	96.76 ± 4.80 b	196.49 ± 7.68 a
Methyl decanoate	nd	nd	6.12 ± 0.14
Ethyl dodecanoate	14.93 ± 0.90 b	8.74 ± 0.50 c	18.35 ± 1.24 a
Ethyl-2-hydroxy-4-methylpentanoate	40.65 ± 0.57 b	76.86 ± 3.65 a	40.38 ± 1.06 b
Ethyl-2-hydroxypropanoate	622.32 ± 11.40 a	572.38 ± 15.28 b	364.21 ± 8.10 c
Ethyl-3-hydroxybutanoate	22.94 ± 0.74 c	44.27 ± 2.24 b	55.29 ± 2.00 a
Ethyl-4-hydroxybutanoate	283.38 ± 3.00 c	434.84 ± 23.11 a	353.89 ± 15.30 b
Ethyl-9-decanoate	132.58 ± 5.34 a	126.48 ± 2.72 a	128.17 ± 2.27 a
Ethyl-9-hexadecenoate	13.28 ± 0.71 b	23.83 ± 2.41 a	24.95 ± 1.59 a
Ethyl hydrogen succinate	278.97 ± 11.75 c	795.38 ± 69.54 a	522.43 ± 24.67 b
Ethyl cinnamate	64.64 ± 4.11 a	nd	61.52 ± 1.53 a
*trans*-3-hexen-1-ol acetate	93.69 ± 3.09 b	914.22 ± 31.67 a	9.37 ± 1.26 c
*cis*-3-hexen-1-ol acetate	1.12 ± 0.34 b	2.66 ± 0.26 a	nd
Ethyl heptanoate	nd	21.13 ± 0.50	nd
Ethyl hexadecanoate	38.66 ± 0.68 a	36.61 ± 0.87 b	39.98 ± 0.72 a
Ethyl-3-hydroxyhexanoate	nd	nd	3.87 ± 0.12
Isoamyl acetate	nd	16.22 ± 0.33 a	0.23 ± 0.06 b
Ethyl nonanoate	52.84 ± 1.34 a	52.77 ± 1.52 a	53.29 ± 0.91 a
3-methylbuthyl ocatnoate	8.35 ± 0.42 b	14.40 ± 1.69 a	11.58 ± 1.19 a
Ethyl octanoate	244.15 ± 5.57 b	779.85 ± 20.57 a	758.85 ± 30.17 a
Methyl octanoate	17.19 ± 1.60 b	nd	19.96 ± 0.66 a
3-methylbutyl pentadecanoate	7.41 ± 0.23 b	9.16 ± 0.12 a	9.74 ± 0.43 a
Ethyl pentadecanoate	31.10 ± 0.86 c	61.59 ± 2.89 a	40.56 ± 2.47 b
2-methyl-propanoate	4002.09 ± 92.78 a	2908.13 ± 120.07 b	1645.54 ± 27.98 c
Butyl ethyl succinate	55.95 ± 3.69 c	94.18 ± 4.13 b	112.44 ± 3.63 a
Ʃ Esters	8563.98 ± 79.48 b	11136.77 ± 86.63 a	7690.21 ± 61.69 c
4-ethyl guaiacol	nd	17.67 ± 1.41 a	14.38 ± 0.67 b
4-ethyl phenol	nd	96.27 ± 2.61	nd
Eugenol	nd	nd	53.76 ± 1.06
Ʃ Volatile phenols	0 ± 0	113.94 ± 2.09 a	68.13 ± 1 b
Benzaldehyde	58.03 ± 2.23 b	566.09 ± 18.47 a	42.10 ± 1.66 b
Benzyl alcohol	422.79 ± 21.06 b	1610.89 ± 147.13 a	521.31 ± 15.57 b
Furfuryl alcohol	nd	nd	150.06 ± 10.41
Furfural	nd	260.99 ± 9.82	nd
2,4-dimethyl-3-pentanol	79.26 ± 4.26 b	nd	112.28 ± 8.88 a
6-methyl-5-hepten-2-one	3.21 ± 0.07 b	9.85 ± 0.17 a	11.93 ± 1.90 a
Acetoin	14.42 ± 1.67 b	nd	22.24 ± 2.49 a
Methionol	53.23 ± 3.53 c	2080.22 ± 45.18 b	5978.68 ± 107.43 a
Ʃ Other compounds	630.95 ± 22.45 c	4528.04 ± 162.84 b	6838.61 ± 98.14 a

Values are means with standard deviations of three separate repetitions. Values with different letter (a, b, c) in the same row are significantly different, according to the Fisher’s LSD test (*p <* 0.05). nd—not detected.

**Table 4:** Polyhenolic compounds in Plavac Mali wines produced sequential L, conventional S and spontaneous N fermentation expressed as (mg/L).

Compound (mg/L)	L	S	N
Caftaric acid	27.01 ± 1.34 b	27.98 ± 1.32 b	35.12 ± 0.59 a
Caffeic acid	9.56 ± 1.15 a	10.31 ± 0.97 a	11.74 ± 1.18 a
Ʃ HCA	36.57 ± 2.49 b	38.28 ± 2.27 b	46.86 ± 1.41 a
Resveratrol 3-*O*-glucoside	7.18 ± 0.28 b	7.80 ± 0.33 ab	8.08 ± 0.17 a
Ʃ Stilbenes	7.18 ± 0.28 b	7.80 ± 0.33 ab	8.08 ± 0.17 a
Gallic acid	31.11 ± 0.25 b	34.25 ± 1.16 a	34.52 ± 1.00 a
Protocatechuic acid	3.93 ± 0.05 a	3.82 ± 0.15 a	3.89 ± 0.08 a
Vanillic acid	5.43 ± 0.26 a	5.11 ± 0.37 a	5.53 ± 0.28 a
Syringic acid	11.42 ± 0.23 b	13.50 ± 0.31 a	11.99 ± 0.10 b
Ʃ HBA	51.90 ± 0.61 b	56.69 ± 1.19 a	55.93 ± 1.09 a
Delphinidin-3-*O*-glucoside	3.45 ± 0.17 c	4.19 ± 0.12 b	4.69 ± 0.15 a
Petunidin-3-*O*-glucoside	8.47 ± 0.15 b	9.80 ± 0.11 a	10.10 ± 0.33 a
Peonidin-3-*O*-glucoside	5.63 ± 0.37 b	7.22 ± 0.14 a	7.26 ± 0.11 a
Malvidin-3-*O*-glucoside	70.32 ± 0.69 b	85.30 ± 2.80 a	82.05 ± 1.79 a
Cyanidin-3-*O*-glucoside	0.55 ± 0.21 a	0.29 ± 0.02 a	0.54 ± 0.16 a
Malvidin-3-(6-*O*-acetyl) glucoside	4.69 ± 0.13 b	5.66 ± 0.29 a	5.78 ± 0.13 a
Malvidin-3-*O*-glucoside-cafeoil-g	0.53 ± 0.03 c	0.73 ± 0.05 b	0.83 ± 0.02 a
Peonidin-3-*O*-(*p*-coumaroyl)-glucoside	0.20 ± 0.06 b	0.67 ± 0.10 a	0.64 ± 0.02 a
Malvidin-3-*O*-(*p*-coumaroyl)-glucoside	8.78 ± 0.25 b	10.31 ± 0.20 a	10.16 ± 0.27 a
Ʃ Anthocyanins	102.61 ± 0.78 b	124.16 ± 2.73 a	122.06 ± 2.20 a
Myricetin-3-*O*-glucoside	4.54 ± 0.22 b	5.42 ± 0.12 a	5.44 ± 0.25 a
Myricetin-3-*O*-glucuronide	0.72 ± 0.07 b	0.60 ± 0.08 b	1.02 ± 0.06 a
Quercetin-3-*O*-galactoside	0.20 ± 0.04 b	0.32 ± 0.03 a	0.31 ± 0.01 a
Quercetin-3-*O*-glucoside	31.79 ± 1.36 b	35.79 ± 0.64 a	35.20 ± 0.75 a
Quercetin-3-*O*-glucuronide	0.75 ± 0.04 b	0.95 ± 0.05 a	0.74 ± 0.05 b
Kaempferol-3-*O*-glucoside	2.83 ± 0.12 a	3.03 ± 0.06 a	2.86 ± 0.11 a
Kaempferol-3-*O*-glucuronide	1.99 ± 0.02 b	2.19 ± 0.03 a	2.11 ± 0.07 a
Isorhamnetin-3-*O*-glucoside	0.18 ± 0.01 b	0.22 ± 0.02 a	0.20 ± 0.02 ab
Ʃ Flavonols	43.00 ± 1.35 b	48.51 ± 0.63 a	47.87 ± 1.28 a
EGCG	9.09 ± 0.33 a	9.87 ± 0.36 a	9.88 ± 0.30 a
ECG	13.23 ± 0.18 b	16.02 ± 0.24 a	13.57 ± 0.20 b
Gallocatechin	59.78 ± 1.19 b	63.62 ± 2.48 ab	65.71 ± 0.93 a
Epigallocatechin	14.17 ± 0.15 b	13.60 ± 0.15 c	15.25 ± 0.28 a
Catechin	25.12 ± 0.56 b	30.35 ± 1.24 a	29.24 ± 0.67 a
Epicatechin	10.56 ± 0.30 b	12.87 ± 0.07 a	13.21 ± 0.56 a
PB1	61.21 ± 2.46 c	98.34 ± 2.31 b	112.76 ± 5.00 a
PB2	5.58 ± 0.10 b	6.79 ± 0.49 a	6.54 ± 0.17 a
PB3	2.48 ± 0.01 c	2.94 ± 0.07 b	3.29 ± 0.10 a
PB4	8.60 ± 0.16 c	10.52 ± 0.56 b	11.96 ± 0.01 a
PA1	1.69 ± 0.03 b	1.94 ± 0.07 a	1.73 ± 0.05 b
Ʃ Flavan3-ols	211.52 ± 3.53 c	266.87 ± 3.93 b	283.15 ± 5.44 a

Values are means with standard deviations of three separate repetitions. Values with different letter (a, b, c) in the same row are significantly different, according to the Fisher’s LSD test (*p <* 0.05).

## Data Availability

Data is contained within the article.

## References

[1] Capozzi V., Garofalo C., Chiriatti M.A., Grieco F., Spano G. (2015). Microbial terroir and food innovation: The case of yeast biodiversity in wine. Microbiol. Res..

[2] Bokulich N.A., Thorngate J.H., Richardson P.M., Mills D.A. (2014). Microbial biogeography of wine grapes is conditioned by cultivar, vintage, and climate. Proc. Natl. Acad. Sci. USA.

[3] Fleet H.G. (2008). Wine yeasts for the future. FEMS Yeast Res..

[4] Han X., Xin Q., Yang S., Li R., Huang W. (2021). Study on the diversity of non-*Saccharomyces* yeasts in Chinese wine regions and their potential in improving wine aroma by *β*-glucosidase activity analyses. Food Chem..

[5] Martins G., Vallance J., Mercier A., Albertin W., Stamatopoulos P., Rey P., Lonvaud A., Masneuf-Pomarède I. (2014). Influence of the farming system on the epiphytic yeasts and yeast-like fungi colonizing grape berries during the ripening process. Int. J. Food Microbiol..

[6] Shekhawat K., Porter T.J., Bauer F.F., Setati M.E. (2018). Employing oxygen pulses to modulate *Lachancea thermotolerans*–*Saccharomyces cerevisiae* Chardonnay fermentations. Ann. Microbiol..

[7] Hranilovic A., Li S., Boss P.K., Bindon K., Ristic R., Grbin P.R., Jiranek V. (2018). Chemical and sensory profiling of Shiraz wines co-fermented with commercial non-*Saccharomyces* inocula. Aust. J. Grape Wine Res..

[8] Molina A.M., Swiegers J.H., Varela C., Pretorius I.S., Agosin E. (2007). Influence of wine fermentation temperature on the synthesis of yeast-derived volatile aroma compounds. Appl. Microbiol. Biotechnol..

[9] Varela C., Siebert T., Cozzolino D., Rose L., McLean H., Henschke P.A. (2009). Discovering a chemical basis for differentiating wines made by fermentation with ‘wild’ indigenous and inoculated yeasts: Role of yeast volatile compounds. Aust. J. Grape Wine Res..

[10] Chatonnet P., Dubourdieu D., Boidron J.N., Lavigne V. (1993). Synthesis of volatile phenols by *Saccharomyces cerevisiae* in wines. J. Sci. Food Agric..

[11] Hranilovic A., Albertin W., Capone D.L., Gallo A., Grbin P.R., Danner L., Bastian S.E., Masneuf-Pomarede I., Coulon J., Bely M. (2021). Impact of *Lachancea thermotolerans* on chemical composition and sensory profiles of Merlot wines. Food Chem..

[12] Jagatić Korenika A.-M.J., Tomaz I., Preiner D., Lavrić M., Šimić B., Jeromel A. (2021). Influence of *L. thermotolerans* and *S. cerevisiae* commercial yeast sequential inoculation on aroma composition of red wines (*cv* Trnjak, Babic, Blatina and Frankovka). Fermentation.

[13] Gobbi M., Comitini F., Domizio P., Romani C., Lencioni L., Mannazzu I., Ciani M. (2013). *Lachancea thermotolerans* and *Saccharomyces cerevisiae* in simultaneous and sequential co-fermentation: A strategy to enhance acidity and improve the overall quality of wine. Food Microb..

[14] Escribano-Viana R., Portu J., Garijo P., López R., Santamaría P., López-Alfaro I., Gutiérrez A.R., González-Arenzana L. (2019). Effect of the sequential inoculation of non-*Saccharomyces*/*Saccharomyces* on the anthocyans and stilbenes composition of Tempranillo wines. Front. Microbiol..

[15] Benucci I., Cerreti M., Liburdi K., Nardi T., Vagnoli P., Ortiz-Julien A., Esti M. (2018). Pre-fermentative cold maceration in presence of non-*Saccharomyces* strains: Evolution of chromatic characteristics of Sangiovese red wine elaborated by sequential inoculation. Food Res. Int..

[16] Porter T.J., Divol B., Setati M.E. (2019). Investigating the biochemical and fermentation attributes of *Lachancea* species and strains: Deciphering the potential contribution to wine chemical composition. Int. J. Food Microbiol..

[17] Gunata Y.Z., Bitteur S., Brillouet J.M., Bayonove C.L., Cordonnier R.E. (1988). Sequential enzymic hydrolysis of potentially aromatic glycosides from grape. Carbohyd. Res..

[18] Escott C., Del Fresno J.M., Loira I., Morata A., Tesfaye W., del Carmen González C., Suárez-Lepe J.A. (2018). Formation of polymeric pigments in red wines through sequential fermentation of flavanol-enriched musts with non-*Saccharomyces* yeasts. Food Chem..

[19] Benito Á., Calderón F., Benito S. (2017). The combined use of *Schizosaccharomyces pombe* and *Lachancea thermotolerans*—Effect on the anthocyanin wine composition. Molecules.

[20] Casassa L.F., Beaver C.W., Mireles M., Larsen R.C., Hopfer H., Heymann H., Harbertson J.F. (2013). Influence of fruit maturity, maceration length, and ethanol amount on chemical and sensory properties of Merlot wines. Am. J. Enol. Vitic..

[21] Kontoudakis N., Esteruelas M., Fort F., Canals J.M., Zamora F. (2011). Use of unripe grapes harvested during cluster thinning as a method for reducing alcohol content and pH of wine. Aust. J. Grape Wine Res..

[22] Van Leeuwen C., Seguin G. (2006). The concept of *terroir* in viticulture. J. Wine Res..

[23] Jagatić Korenika A.-M., Tomaz I., Preiner D., Plichta V., Jeromel A. (2021). Impact of commercial yeasts on phenolic profile of Plavac Mali wines from Croatia. Fermentation.

[24] Winkler A.J., Cook J.A., Kliewer W.M., Lider L.A. (1974). General Viticulture.

[25] Karoglan M., Prtenjak M.T., Šimon S., Osrečak M., Anić M., Kontić J.K., Andabaka Ž., Tomaz I., Grisogono B., Belušić A. Classification of Croatian winegrowing regions based on bioclimatic indices. Paper presented at the E3S Web of Conferences, XIIth International Terroir Congress.

[26] OIV (2016). Compendium of International Methods of Wine and Must Analysis.

[27] Šikuten I., Štambuk P., Karoglan Kontić J., Maletić E., Tomaz I., Preiner D. (2021). Optimization of SPME-Arrow-GC/MS method for determination of free and bound volatile organic compounds from grape skins. Molecules.

[28] Song N.E., Lee J.Y., Lee Y.Y., Park J.D., Jang H.W. (2019). Comparison of headspace-SPME and SPME-Arrow-GC-MS methods for the determination of volatile compounds in Korean salt-fermented fish sauce. Appl. Biol. Chem..

[29] Tomaz I., Maslov L. (2016). Simultaneous determination of phenolic compounds in different matrices using phenyl-hexyl stationary phase. Food Anal. Methods.

[30] Berente B., De La Calle García D., Reichenbächer M., Danzer K. (2000). Method development for the determination of anthocyanins in red wines by high-performance liquid chromatography and classification of German red wines by means of multivariate statistical methods. J. Chromatogr. A.

[31] Genovese A., Dimaggio R., Lisanti M.T., Piombino P., Moio L. (2005). Aroma composition of red wines by different extraction methods and gas chromatography-SIM/MASS spectrometry analysis. Ann. Chim..

[32] Loscos N., Hernandez-Orte P., Cacho J., Ferreira V. (2007). Release and formation of varietal aroma compounds during alcoholic fermentation from nonfloral grape odorless flavor precursors fractions. J. Agric. Food Chem..

[33] Genovese A., Caporaso N., Moio L. (2021). Influence of yeast strain on odor-active compounds in Fiano wine. Appl. Sci..

[34] Carrau F.M., Medina K., Boido E., Farina L., Gaggero C., Dellacassa E., Versini G., Henschke P.A. (2005). De novo synthesis of monoterpenes by *Saccharomyces cerevisiae* wine yeasts. FEMS Microbiol. Lett..

[35] Keyzers R.A., Boss P.K. (2010). Changes in the volatile compound production of fermentations made from musts with increasing grape content. J. Agric. Food Chem..

[36] Cameleyre M., Lytra G., Tempere S., Barbe J.C. (2015). Olfactory impact of higher alcohols on red wine fruity ester aroma expression in model solution. J. Agric. Food Chem..

[37] De-La-Fuente-Blanco A., Sáenz-Navajas M.P., Ferreira V. (2016). On the effects of higher alcohols on red wine aroma. Food Chem..

[38] Guth H. (1997). Quantitation and sensory studies of character impact odorants of different white wine varieties. J. Agric. Food Chem.

[39] Nan L., Liu L., Li Y., Huang J., Wang Y., Wang C., Wang Z., Xu C. (2021). Comparison of aroma compounds in Cabernet Sauvignon red wines from five growing regions in Xinjiang in China. J. Food Qual..

[40] Balikci E.K., Tanguler H., Jolly N.P., Erten H. (2016). Influence of *Lachancea thermotolerans* on cv. Emir wine fermentation. Yeast.

[41] Delgado J.A., Sánchez-Palomo E., Alises M.O., Viñas M.G. (2022). Chemical and sensory aroma typicity of La Mancha Petit Verdot wines. LWT.

[42] Romano P., Braschi G., Siesto G., Patrignani F., Lanciotti R. (2022). Role of yeasts on the sensory component of wines. Foods.

[43] Barbosa C., Mendes-Faia A., Mendes-Ferreira A. (2012). The nitrogen source impacts major volatile compounds released by *Saccharomyces cerevisiae* during alcoholic fermentation. Int. J. Food Microbiol..

[44] Qian X., Liu Y., Zhang G., Yan A., Wang H., Wang X., Pan Q., Xu H., Sun L., Zhu B. (2019). Alcohol acyltransferase gene and ester precursors differentiate composition of volatile esters in three interspecific hybrids of *Vitis labrusca*× *V. vinifera* during berry development period. Food Chem..

[45] Fairbairn S., McKinnon A., Musarurwa H.T., Ferreira A.C., Bauer F.F. (2017). The impact of single amino acids on growth and volatile aroma production by *Saccharomyces cerevisiae* strains. Front. Microbiol..

[46] Reyes-Sánchez F.J., Páez-Lerma J.B., Rojas-Contreras J.A., López-Miranda J., Soto-Cruz N.Ó., Reinhart-Kirchmayr M. (2019). Study of the enzymatic capacity of *Kluyveromyces marxianus* for the synthesis of esters. Microb. Physiol..

[47] Mendes-Ferreira A., Barbosa C., Falco V., Leão C., Mendes-Faia A. (2009). The production of hydrogen sulphide and other aroma compounds by wine strains of *Saccharomyces cerevisiae* in synthetic media with different nitrogen concentrations. JIMB.

[48] Contreras-Ruiz A., Alonso-del-Real J., Barrio E., Querol A. (2023). *Saccharomyces cerevisiae* wine strains show a wide range of competitive abilities and differential nutrient uptake behavior in co-culture with *S. kudriavzevii*. Food Microbiol..

[49] Ferreira V., López R., Cacho J.F. (2000). Quantitative determination of the odorants of young red wines from different grape varieties. J. Sci. Food Agric..

[50] Silva R., Coelho E., Aguiar T.Q., Domingues L. (2021). Microbial biosynthesis of lactones: Gaps and opportunities towards sustainable production. Appl. Sci..

[51] Chou H.-C., Šuklje K., Antalick G., Schmidtke L.M., Blackman J.W. (2018). Late-season Shiraz berry dehydration that alters composition and sensory traits of wine. J. Agric. Food Chem..

[52] Morata A., Loira I., González C., Escott C. (2021). Non-*Saccharomyces* as biotools to control the production of off-flavors in wines. Molecules.

[53] Coghe S., Benoot K., Delvaux F., Vanderhaegen B., Delvaux F.R. (2004). Ferulic acid release and 4-vinylguaiacol formation during brewing and fermentation: Indications for feruloyl esterase activity in *Saccharomyces cerevisiae*. J. Agric. Food Chem..

[54] Weldegergis B.T., Crouch A.M., Górecki T., De Villiers A. (2011). Solid phase extraction in combination with comprehensive two-dimensional gas chromatography coupled to time-of-flight mass spectrometry for the detailed investigation of volatiles in South African red wines. Anal. Chim. Acta..

[55] Carrasco-Sánchez V., John A., Marican A., Santos L.S., Laurie V.F. (2015). Removal of 4-ethylphenol and 4-ethylguaiacol with polyaniline-based compounds in wine-like model solutions and red wine. Molecules.

[56] Perestrelo R.M.D.S., Fernandes A., Albuquerque F.F., Marques J.C., Câmara J.D.S. (2006). Analytical characterization of the aroma of Tinta Negra Mole red wine: Identification of the main odorants compounds. Anal. Chim. Acta.

[57] Monagas M., Bartolomé B., Gómez-Cordovés C. (2005). Evolution of polyphenols in red wines from *Vitis vinifera* L. during aging in the bottle. Eur. Food Res. Technol..

[58] Razmkhab S., Lopez-Toledano A., Ortega J.M., Mayen M., Merida J., Medina M. (2002). Adsorption of phenolic compounds and browning products in white wines by yeasts and their cell walls. J. Agric. Food Chem..

[59] De Melo Lopes L.M., Costa Batista L.H., Gouveia M.J., Leite T.C.C., de Mello M.R.F., de Assis S.A., de Sena A.R. (2018). Kinetic and thermodynamic parameters, and partial characterization of the crude extract of tannase produced by *Saccharomyces cerevisiae* CCMB 520. Nat. Prod. Res..

[60] Ferrer-Gallego R., Hernández-Hierro J.M., Rivas-Gonzalo J.C., Escribano-Bailón M.T. (2014). Sensory evaluation of bitterness and astringency sub-qualities of wine phenolic compounds: Synergistic effect and modulation by aromas. Food Res. Int..

[61] Morata A., Gómez-Cordovés M.C., Calderón F., Suárez J.A. (2006). Effects of pH, temperature and SO2 on the formation of pyranoanthocyanins during red wine fermentation with two species of *Saccharomyces*. Int. J. Food Microbiol..

[62] Schwarz M., Wabnitz T.C., Winterhalter P. (2003). Pathway leading to the formation of anthocyanin−vinylphenol adducts and related pigments in red wines. J. Agric. Food Chem..

[63] Cerpa-Calderón F.K., Kennedy J.A. (2008). Berry integrity and extraction of skin and seed proanthocyanidins during red wine fermentation. J. Agric. Food Chem..

